# Effectiveness of Psychomotor Therapy among Children with Graphomotor Impairment with and without DCD-Diagnosis

**DOI:** 10.3390/children10060964

**Published:** 2023-05-29

**Authors:** Sibylle Hurschler Lichtsteiner, Melanie Nideröst, Carlo Di Brina, Christian Marquardt, Stefanie Wyss, Alois Buholzer, Werner Wicki

**Affiliations:** 1 Languages Research Group, University of Teacher Education Lucerne, CH-6003 Lucerne, Switzerland; 2 Institute for Educational Support for Behaviour, Social-Emotional, and Psychomotor Development, University of Teacher Education in Special Needs Zurich, CH-8050 Zurich, Switzerland; 3 Department of Human Neuroscience, Sapienza University of Rome, I-00185 Rome, Italy; 4 Science&Motion GmbH, D-81547 Munich, Germany; 5 Institute for Diversity in Education, University of Teacher Education Lucerne, CH-6003 Lucerne, Switzerland

**Keywords:** handwriting, developmental coordination disorder, dysgraphia, psychomotor therapy, effectiveness of psychomotor therapy

## Abstract

In Switzerland, psychomotor therapy (PMT) is a standard treatment for children with graphomotor impairments, but scientific evidence of its effectiveness is rare. To investigate the effectiveness of PMT, we conducted a randomised field trial (RFT). The sample consisted of 121 first and second graders with graphomotor impairments, some of whom met the criteria of developmental coordination disorder, while the remaining suffered from developmental dysgraphia. The treatments lasted over 5 months. Handwriting fluency and consistency were measured five times on a digitising tablet. All participating children completed a self-concept interview, and a standardised fine motor performance test twice. Psychomotor therapy significantly improved the fine motor skills of the therapy group compared to those of the waiting group. However, there was no evidence that the treated children improved more than the waiting children in terms of their graphomotor skills such as frequency, automaticity, and consistency of forming letters. Finally, the children of the therapy group showed partial improvements in their handwriting self-concept, while those of the waiting group children remained stable. This short-term RFT demonstrated the effectiveness of PMT in terms of fine motor skills and some aspects of the handwriting self-concept but showed no effects on handwriting fluency and consistency.

## 1. Introduction

### 1.1. Handwriting Research in Children

Current scientific models conceptualise handwriting as a complex neuromotor skill, which involves both cognitive and motor processes that are at least partially executed synchronically in a parallel mode [1,2,3]. Furthermore, the interferences between central processes and ongoing movement executions revealed in recent studies demonstrate that handwriting functions in a cascading manner [4,5,6,7,8,9]. As soon as the child learns to write by hand, he or she has to acquire the allographs of the respective language, the corresponding phoneme sounds, and the hand and finger movements needed to execute the allographs. Subsequently, the acquisition of spelling and orthographic coding, as well as the execution of handwriting movements improve continuously to become automated, fluent, and efficient [10,11,12]. Although developmental increases in handwriting speed are well documented [13], research on the development of handwriting fluency in terms of automaticity is less prominent (for exceptions, see e.g., [14,15]). 

Novice writers slowly write single movements with visual control (i.e., feedback control), and their speed profile shows constant acceleration and deceleration pulses [16]. Due to the physiological limitation of eye tracking movements, this is possible, at most, up to a frequency of approximately 2 Hz [17]. On the contrary, skilled writers show only one movement pulse per stroke unit. In analysis, automated movement patterns show a smooth, regular velocity profile with only one velocity reversal per upward and downward stroke. Familiar patterns, allographs, and words are expected to be performed by older children and adults through this much more automated process (feed-forward control) [18,19,20,21]. Advanced handwriting fluency, in terms of increased stroke frequency and automaticity, has been shown to be an important prerequisite for speed in third and fourth graders [15].

### 1.2. Developmental Coordination Disorder and Handwriting Difficulties

According to the Diagnostic and Statistical Manual of Mental Disorders (DSM-5) [22] and the International clinical practical guidelines [23], developmental coordination disorder (DCD) is diagnosed in children suffering from significant difficulties with motor and fine motor coordination that are not due to medical or neurological conditions (e.g., muscular dystrophy or cerebral palsy), or intellectual disabilities.

To be diagnosed, the disorder must severely limit the activities of daily living and/or academic achievement, with a negative impact on activities, such as dressing, feeding, and catching a ball, as well as on fine motor movements, such as handwriting, although handwriting problems can affect academic success in many ways.

Similarly, the International Classification of Diseases ICD [24] characterizes DCD as “a significant delay in the acquisition of gross and fine motor skills and impairment in the execution of coordinated motor skills that manifest in clumsiness, slowness, or inaccuracy of motor performance”. According to the ICD-10, the child’s motor skills are substantially reduced compared to their typically developing peers, causing significant and persistent limitations in functioning since early childhood. 

Depending on the cut-off criteria used, the estimated prevalence of DCD amounts to between 3% and 10% of all school-aged children. Many children with DCD have co-occurring conditions such as attention deficit and hyperactivity disorder (ADHD) [25], learning difficulties [26], dyslexia [27], or language disorder [28]; however, these conditions do not exclude the DCD diagnosis. Moreover, DCD can co-occur with pervasive developmental disorders (autistic spectrum, Rett syndrome). While the DSM-5 criteria would exclude the DCD diagnosis in this case, the Leeds Consensus Statement [29] argues for a dual diagnosis, given that DCD is a separate neurodevelopmental disorder.

DCD presents with a wide variety of impairments, including affected gross-motor skills, balance problems [30,31], impaired fine motor skills, which emerge among the majority of DCD children at the time when handwriting is acquired [32]. As the proposed research focuses on the therapy of handwriting difficulties among children with DCD, we address these problems in more detail in the following sections. 

According to previous studies, most children with DCD have poor handwriting skills [33,34], particularly poor letter arrangements (i.e., writing less legibly), high spatial variability caused by a lack of control of spatial accuracy [35,36,37], or prominent difficulties relating to the temporal aspect of handwriting [38]. Additionally, children with DCD write fewer letters than non-DCD controls when copying [39], show greater disfluency [40], and spend more time pausing compared to non-DCD controls [41].

### 1.3. Developmental Dysgraphia without DCD

Hamstra-Bletz and Blote [42] defined developmental dysgraphia (DD) as a difficulty in the production of written language mainly related to the mechanics of handwriting. A broader understanding of DD refers to an impairment of the transcription process of writing; for instance, a child with DD struggles more with retrieving and producing letters and words than their typically developing peers [43]. Accordingly, handwriting difficulties in children with DD are not merely due to (fine-) motor processes. Respective difficulties also rely on cognitive processes (internal representation of letter formation and written words, retrieval of respective items from memory, and spelling) as outlined by McCloskey and Rapp [44], and by Berninger [45], who titles handwriting “language by hand” (p. 39). The term developmental dysgraphia refers to a condition in which difficulties arise during the acquisition of handwriting despite opportunities to learn, and in the absence of a known neuropathology or sensory-motor dysfunction. 

Children with DD have a wide range of conditions compared to their typically developing peers, especially the inability to form correct letters [46]. By implementing the Dynamic Time Warping (DTW) method, Di Brina et al. [47] found much higher variability in the consistency of letter forms (defined as “the stability in time and space … while the same letter is repeatedly written” [38] (p. 2037) among poor writers, which was independent of other kinematic results of, for instance, larger trajectories or faster movements. Interestingly, the deviation decreased when the children were asked to write faster. Additionally, lack of legibility, dysfluent handwriting [48], erasing and overwriting of letters [49], lack of endurance and velocity [50], and exerting too much pressure on the paper are often found among children with DD. 

Prunty and Barnett [46] reported very similar findings for DD and DCD, but did not discover a reduced handwriting speed in children with DD and DCD; however, they did report that both groups paused for a longer time during free writing compared to the typically developing controls. One reason for the longer pauses could be a strong spelling-motor interaction in children with DD given that difficulties with spelling automaticity negatively affect word writing [51].

In a Swiss study among 60 children attending school-based PMT and suffering from graphomotor difficulties [52,53], only 46% of the involved children fulfilled the cut-off criteria for DCD (M-ABC-2 below the 16th percentile) with respect to motor impairment, which is consistent with the results of Overvelde and Hulstijn [54], who found 17% of the Dutch second graders to be dysgraphic, a prevalence that is much higher than that for DCD.

### 1.4. Consequences of Poor Handwriting

Irrespective of its aetiology, poor handwriting has a negative impact on the physical and emotional well-being of children [49] as well as on their academic achievement (e.g., as poor handwriting is slow, these children have difficulty completing academic tasks within a given time). According to Graham et al. [55], lower-order skills (transcription) interfere with higher-order skills (translational processes); thus, children with poor handwriting compose shorter and worse texts compared to their typically developing peers [56]. Even if the text quality is comparable, low legibility is associated with worse ratings of text quality by teachers [57].

### 1.5. Therapeutic Approaches for Children with DCD

Over the last 15 years, several evidence-based interventions for children with DCD have been developed and published, mostly focusing on the training of specific tasks [58].

Neuromotor task training (NTT) is related to motor learning theories and focuses on the interaction between the child, task, and environment. The child choses their preferred task, the therapist teaches via observation, precise feedback, and appropriate variation of the task and environment [59,60]. The Cognitive Orientation to Daily Occupational Performance programme (CO-OP) is another approach that has been shown to be successful by using cognitive problem-solving strategies to learn targeted skills [61]. The cognitive motor approach of Henderson and Sugden [62] has been extended by including the educational systems around the child and is termed ecological intervention (EI). Furthermore, there is some evidence that neuropsychological treatments like motor imaginary training [63] help to develop the automatisation of movements.

### 1.6. Therapeutic Approaches for Children with Handwriting Difficulties

According to the current German–Austrian–Swiss health care guidelines on the definition, diagnosis, treatment, and psychosocial aspects of circumscribed developmental disorders of motor functions [64], therapies should be based on individualised planning. In addition, for severe graphomotor impairment, a medical examination is necessary to exclude neurological diseases, such as tremors, that might cause similar problems, and to decide whether direct task training is purposeful. Notably, the therapy should help children to cope with the tasks of daily life; therefore, the training must focus on what is important for the child and their family. 

In the case of graphomotor development, parents and teachers mostly recognise the importance of handwriting, but place too much emphasis on neatness [65]. Broader objectives should be improvements in movement skills, including an increase in handwriting fluency and the automaticity of written letterforms, increased self-confidence, and motivation for written communication, and to play and express oneself by painting, drawing, and writing [66,67]. The individualised setting of reachable goals and the self-evaluation of the process are efficient features of therapy title [68] and [67] (pp. 41–44). It is important to involve teachers to establish commitment and identify appropriate methods for both schools and therapy [69].

As this study investigates the situation in the German-speaking part of Switzerland, PMT should be briefly explained.

PMT is influenced by different approaches and theoretical concepts [70] (p. 32). According to Eggert and Lütje-Klose [71], among the hypotheses of therapeutic effects, because of the training of the targeted skills, not only the direct improvement has to be considered, but also the transfer effects due to the improvement in executive functions and the children’s experiences of stabilisation in their personality owing to improved self-confidence after successful motor learning. Therefore, the therapists often prefer the child-centred approach described by Zimmer [72], which places an emphasis on the improvement of competencies and self-competence [73]. Regarding handwriting, graphomotor skills are trained to allow children to express themselves by drawing and writing [74]. In Switzerland, first graders learn a print letter alphabet and are taught to develop their own personal fluent and legible handwriting over the following years [75] based on evidence-based teaching materials, which is also partly used in therapy. Progress in better-automated, more fluent, and more legible handwriting may also improve achievement at school, largely because better automated skills use less working memory capacities and more resources are released for text production [76].

PMT is part of the school-based services for children with special needs provided by the cantonal ministries of education. In contrast to medical therapeutic measures, PMT is generally paid for by the state, and not health insurance companies; therefore, there is no need to obtain a medical diagnosis of DCD in advance. Therapists understand themselves as being part of the educational system and cooperate regularly with teachers [77]. Children usually attend PMT in a specialised room close to their classroom, and in some cases, the therapist will also work in the classroom. Sessions are scheduled during school time, in single settings or in small groups [78]. 

In summary, PMT is well-known and widely accepted in the field of Swiss special needs education, but only a few studies of its effectiveness have been conducted [79,80]. In particular, there is a lack of scientific evidence with respect to the effectiveness of PMT in treating the handwriting difficulties of children with DCD or DD despite its nationwide school-based provision in Switzerland. Conversely, several foreign studies have considered the effectiveness of occupational therapy among children with DCD [81]. Therefore, we conducted an experimental field study to investigate the probable improvements of fine motor and handwriting skills among children with DCD and DD attending a 16-week course of PMT and compared their results to those of a waiting group. With respect to handwriting, we focused on the course characteristics of the respective improvements, including the consistency of written letters and dynamic patterns, such as the automaticity and fluency of handwriting.

According to the theoretical considerations outlined above, we examined the following hypotheses: (1) PMT for children with DCD and DD improves their fine motor skills compared to the children in the control group; (2) PMT for children with DCD and DD continually improves their handwriting skills with respect to the frequency of up and down strokes, automaticity, and consistency of forming letters compared to the children in the control group; and (3) PMT for children with DCD and DD continually improves their handwriting self-concept compared to the children in the control group.

## 2. Materials and Methods

### 2.1. Design

**Figure 1:**
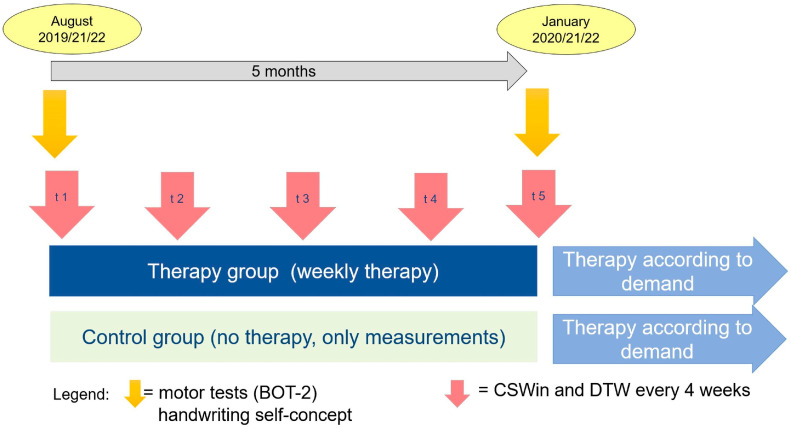
Study design.

To compare the developmental course of children with and without therapy, we employed a randomised waiting-control-group design with pre-, peri-, and post-test measurements. The sample included a therapy group (children attending therapy) and a control group (children waiting for therapy). The participants were first and second graders (*n* = 121) who were randomly assigned to the therapy or control group (Figure 1). Handwriting process measures were assessed five times, while self-concept and fine motor skills were only assessed twice, at pre- and post-test. Over a 3-year period, we examined three independent cohorts. The assessments for each cohort started in August and ended in the following February; therefore, we studied the effectiveness of the treatments over 16 weeks.

The children of the therapy group attended between 9 and 18 therapy sessions (M = 12.7) carried out by experienced psychomotor therapists, with each session lasting 45 min. The therapists reported their interventions using a structured therapy protocol [53,82]. 

The children of the control group did not attend any therapy sessions, or any other service related to fine motor training, but received the usual handwriting lessons at school.

In February, after the end of the study, the children of the control group were also made it possible to start therapy. Regarding the therapy group, the therapists decided on the continuation of the therapy depending on the needs of the particular child.

### 2.2. Participants

All participating children (*n* = 121) were recruited by psychomotor therapists employed by the city of Zurich. The children had been referred for PMT for the first time due to graphomotor impairments. The sample corresponded to 7.6% of all first and second graders registered in Zurich for a psychomotor assessment because of graphomotor difficulties in the years 2018–2021. To assign the children to the DCD or DD group, the therapists used the German version of the Movement Assessment Battery for Children-2 (M-ABC-2) [83], a translated version of the DCD Questionnaire 2007 (DCDQ’07) [84,85] and anamnestic data. Children assigned to the DCD group met the criteria of ICD-10 yielding an M-ABC-2 score below the 16th percentile, as well as experienced handwriting problems that were serious enough to interfere with their academic performance and social integration. Moreover, their motor performance was poorer than expected given their chronological age and did not arise from neurological disease or mental retardation. A medical check-up was conducted for the children of the DCD group by the medical health services of the City of Zurich or by the families’ paediatrician to guarantee the fulfilment of the ICD-10 exclusion criteria. Children with M-ABC-2 scores on the 16th percentile and greater who also showed graphomotor problems were assigned to the DD group. ADHD or mild learning problems were not used as exclusion criteria. Through a random allocation procedure, the participating children were assigned to the therapy and control groups. Active consent for the participation in the study was given by all parents of the participating children.

The sample consisted of 48 first graders (39.7%) and 73 second graders (60.3%), among whom 74.4% were boys, 87.6% were righthanders, reflecting the general distribution of children attending PMT, 34.7% met the criteria of DCD, and 65.3% showed signs of DD without meeting the criteria of DCD. The proportion of children with DCD was higher among the first graders (58.3%) compared to the second graders (30.1%; χ^2^ = 9.50, df = 1, *p* = 0.002). The mean age measured at t1 was 7 years and 2 months (SD = 7 months, ranging from 6 years to 8 years 9 months) (Table 1). 

During the study, one child broke his arm before t4 and had to be excluded for the fine motor and graphomotor examinations of t4 and t5. One child had to be excluded from the second fine motor test because he had started a medical treatment for ADHD before t5. Six children missed the 2^nd^ fine motor test and the self-concept evaluation due to scheduling problems. 

### 2.3. Material

Handwriting movements were recorded using writing tablets (Wacom Intuos PRO-medium tablet) connected to a notebook. All tasks were written down with the “Wacom Inking Pen” KP130—an induction pen with ballpoint refill. An extended version of the software CSWin DTW [86] with CSWin DTW plugin [87] (Marquardt et al., 2021), with a recording frequency of 200 Hz and an accuracy of 0.1 mm on the x- and y-axes, was used for recording and analysis. For the calculation and smoothing of the velocity and acceleration signals, non-parametric regression methods (kernel estimation) were included in the mathematical calculation procedures of CSWin [88]. 

### 2.4. Procedure

There were 24 trained psychomotor therapists involved, working in the city of Zurich. They treated between one and four children per cohort, whereby some therapists were involved in all three cohorts and others only in one or two cohorts. The therapists did not receive information about the results in between the study cohorts, only at the end. After study completion, an information event was used to collect the therapists’ comments and interpretations on the revealed results. 

The tablet recordings were conducted approximately every 4 weeks by trained university staff. Each child was examined individually in a separate room of the school building or in the therapy room. The child sat beside the test administrator. In front of the child was the Wacom tablet with the special pen and a sheet of paper attached to the surface of the tablet. The sequence of the 20 min examination was predetermined by the pre-programmed task sequence of the software. The original items of CSWin have been extended within several studies [15,53,89] to include tasks that are typical for the stages of handwriting development. All participants performed 15 digital handwriting items in the same order, including basic graphomotor movements (Table 2).

For reasons of efficiency, we limited the statistical analyses to the faster, and usually better, second attempt of basic movements and omitted the first trial and the two difficult patterns (e.g., garlands and double loops).

The basic movements were directly demonstrated by the experimenter and the children were made it possible to try them out on a laminated card; afterwards, the task was performed twice (the second one as fast as possible) to ensure the best possible performance based on the combined visual and tactile-kinaesthetic information. 

With respect to writing repetitive letter sequences, many children were not capable of reproducing valid recognisable letters within the set time. Consequently, we offered (starting with measurement point 3) for the letter trace to first be retraced with the finger on a laminated card with an enlarged a-shape to ensure that the correct sequence could be reproduced successfully without interruption. 

The remaining tasks were presented visually using instruction cards, with ComicSansSerif used as the font for the text. The children were asked to use the writing type that they had learnt at school. Unstructured white paper was used for all items given that, according to Quenzel and Mai [20], visual guidelines have a negative influence on writing speed. Only in the case of the tasks with repetitive letter sequences, was a discrete visual structure in the form of light grey bars provided for measurement purposes. Observations on validity, pen postures, and other difficulties were noted and used to clean the dataset. If a task was solved incorrectly (e.g., if it was aborted too early), a maximum of one repetition was allowed.

### 2.5. Measures

#### 2.5.1. Fine Motor Performance

To measure the fine motor performance, the German version [90] of the Brunininks–Oseretsky Test of Motor Proficiency BOT-2 [91] was administered by the therapists themselves before the first measurement and after the 5th measurement. This is a standardised, norm-referenced, individually administered measure, specifically of fine manual control, manual coordination, body coordination, and strength and agility, which is often used in clinical and school practice settings. For the purpose of this study, subtest 1 (fine motor precision) and subtest 2 (fine motor integration) were used, both of which were combined in the fine manual control scale. The therapists were not blinded with respect to the group assignments, but the tests were blindly evaluated by the research assistant.

#### 2.5.2. Process-Based Handwriting Measures

Velocity: Stroke frequency (FREQ) refers to the number of upward and downward strokes per second. To calculate these strokes, the written trace is divided in subsequent up and down segments by CSWin [92]. This measure seems more appropriate than assessing the absolute writing speed (mm/s) as the speed will directly depend on a person’s individual writing size. Children with handwriting difficulties have been found to perform slowly but to improve steadily throughout PMT [52]. Additionally, the data provide an insight into the level of motor control already achieved; thus, while visually controlled movements show up in a stroke frequency of approximately 2 Hz or lower, values greater than this indicate that handwriting is performed by sufficiently automated movements [17,93].

Automaticity: We measured automaticity by the number of inversions in velocity (NIV). The NIV indicates the average number of velocity changes occurring within writing strokes. In the optimal case, the velocity profile is unimodal (acceleration followed by deceleration), resulting in a value of NIV = 1. A fluent adult handwriter requires nearly one velocity change per stroke (acceleration followed by deceleration), resulting in an NIV score that is close to 1 [88]. Children with handwriting difficulties demonstrate a much higher NIV, indicating a substantial lack of automaticity [52].

Dynamic time warping (DTW): The digital time normalisation DTW is a method used for the pattern comparison of different sequences of values to calculate relative difference measures. The DTW analysis for writing [94,95] compares the spatial and temporal similarity of repeatedly written traces. Di Brina et al. [47] used the DTW method to compare the shape of written letters to analyse the spatial properties of the handwriting of children with writing problems. The calculated DTW distance is the average point-to-point distance between the respective written letters and the individually calculated prototype of this letter with a normated size of 1. To better understand the distribution of the deviation, the percentages of coherent letters (named as percentage coherence, d < 0.05) and deviant letters (named as percentage deviation, d > 0.1) are calculated [86,87].

#### 2.5.3. Handwriting Self-Concept

To assess the handwriting self-concept, an extended version of an instrument we had developed and used previously [53,96]. The children were interviewed by the therapists regarding their self-concept over eight aspects. They were asked whether they considered their handwriting to be nice, fluent, loose, and legible; if they used correct letter sequences; if they felt secure when writing; and if they wrote with joy and were satisfied with the product. The children were asked to use a token that they positioned on a six-step staircase made of building blocks, where the higher the step the more positive their estimation. The therapists noted the answers on the related 6-point scale. Within this study, we used the scale twice, once at the pre-test and once at the post-test.

#### 2.5.4. Therapy Aims and Therapy Protocols

The 61 children attending PMT weekly from August to January were treated according to individual goals set at the beginning. All therapeutic interventions were recorded in terms of content and time by means of a therapy protocol [82]; this yielded between 10 and 18 protocols per child, indicating the interventions chosen per session and the time spent on them. Within each focus area, the therapist indicated the selected sub-areas. The protocol dataset included 671 protocols, referring to comprehensive information on treatment contents and procedures [82].

#### 2.5.5. Data Analysis

All statistical analyses were performed using the Statistical Package for the Social Sciences version 28. For the standardised tests (BOT-2), transformed T-values were used in the analysis. 

To examine the intervention effects on fine motor control, a two-way analysis of variance (ANOVA) using time as a repeated factor, group and diagnosis as between factors, and grade as a covariate was employed. 

Because of the young age of our sample and the resulting difficulties of many children with certain handwriting tasks, the respective data contain a significant number of missing data (indicating that a child was unable to perform the task at this trial). In addition, many of the handwriting process variables were skewed and not normally distributed (particularly the NIV), and therefore did not meet the requirements of the traditional ANOVA approach. Consequently, we calculated generalised estimating equations (GEE), which are designed to handle missing data and are also suitable for non-normal distributed variables given that the missing data were distributed completely at random (MCAR) [97,98]. When the MCAR requirement was not fulfilled (in addition to the non-normal distribution), we decided to perform separate non-parametric tests (Wilcoxon) for the therapy and waiting groups, comparing t1 vs. t5 only. The rationale of the Wilcoxon analyses was to detect a significant change over time (t1 vs. t5) in one group but not in the other, indicating that there was a group difference.

Similarly to the process variables described above, the handwriting self-concept variables were not normally distributed, and the MCAR criterion was not met. Therefore, we employed Wilcoxon tests as described previously.

## 3. Results

### 3.1. Fine Motor Skills

**Figure 2:**
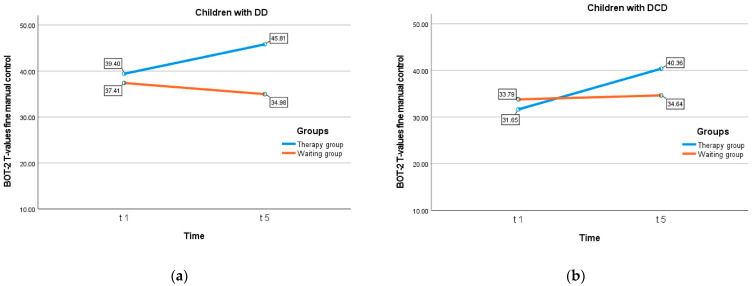
(**a**) Fine manual control (Brunininks–Oseretsky Test of Motor Proficiency BOT-2, T-values) among children with developmental dysgraphia (DD); (**b**) fine manual control (Brunininks–Oseretsky Test of Motor Proficiency BOT-2, T-values) among children with developmental coordination disorder (DCD).

The 2 (group) × 2 (diagnosis) × 2 (time) repeated-measures ANOVA revealed two significant main effects and one significant two-way interaction (Table 3). Firstly, children with DCD performed worse in fine manual control than children with DD. Secondly, the therapy group performed better than the waiting group taking both time points into account. Thirdly, children attending therapy improved in fine manual control over time, while this was not the case among the children of the waiting group who stagnated at the initial level (time × group interaction: F = 28.74, df = 1/108, *p* = 0.000, Eta2 = 0.210). All other main or interaction terms turned out to be non-significant (including the three-way interaction) (Figure 2).

### 3.2. Process-Based Handwriting Results

In the following sections, we analyse how the process-based handwriting measures develop from t1 to t5 in both groups by means of the GEE procedure if the MCAR condition is fulfilled. If this requirement is not met, we analyse by means of nonparametric Wilcoxon tests only taking t1 and t5 into account. The means and standard deviations for all measures from t1 to t5 are reported in Table A1 and Table A2 in Appendix A, as well as missing data due to invalid attempts, which were more prevalent among the basic movements and over the first trials (t1 and t2).

GEE analyses: The GEE analyses revealed several handwriting fluency (FREQ) improvements over time for both groups; this was the case regarding fast finger movements (Wald-Chi2 = 10.5, df = 4, *p* < 0.05), repetitive letter sequences (without speed specification) (Wald-Chi2 = 30.8, df = 4, *p* < 0.001), copying a word (Wald-Chi2 = 101.6, df = 4, *p* < 0.001), and copying a sentence (without speed specification) (Wald-Chi2 = 116.1, df = 4, *p* < 0.001). The same analyses did not reveal any group effects and, with one exception, any group × time interactions. The exception was an unexpected group × time interaction with respect to repetitive letter sequences (without speed specification), indicating more improvement among the waiting group compared to the therapy group (Wald-Chi2 = 10.7, df = 4, *p* < 0.05).

Non-parametric analyses (Wilcoxon): Regarding handwriting fluency (FREQ), we found a time effect for both groups for repetitive letter sequences (as fast as possible) (therapy group: z = −4.41, *p* < 0.001; waiting group: z = −4.23, *p* < 0.001). Only the waiting group but not the therapy group improved from t1 to t5 in wrist movements (fast) (z = −2.532, *p* = 0.011), combined finger and wrist movements when circling (z = −2.621, *p* = 0.009), and repetitive letter sequences (precisely) (z = 4.493, *p* < 0.001).

In contrast, the therapy group improved in automaticity (NIV) in combined finger and wrist movements (fast) (z = −2.634, *p* = 0.008).

Furthermore, with respect to automaticity, both groups improved from time 1 to time 5 in repetitive letter sequences (without speed specification) (therapy group: z = −2.026, *p* = 0.043; waiting group: z = −4.163, *p* < 0.001), repetitive letter sequences (as fast as possible) (therapy group: z = −4.597, *p* < 0.001; waiting group: z = −3.959, *p* < 0.001), repetitive letter sequences (as precisely as possible) (therapy group: z =−2.121, *p* = 0.034; waiting group: z = −4.289, *p* < 0.001), in copying a word (therapy group: z = −4.030, *p* < 0.001; waiting group: z = −5.103, *p* < 0.001), in copying a sentence without speed specification (therapy group: z = −5.278, *p* < 0.001; waiting group: z = −5.530, *p* < 0.001), and under fast condition (therapy group: z = −3.818, *p* < 0.001; waiting group: z = 4.803, *p* < 0.001).

Regarding the measures of DTW, for the criteria of distance, consistency, and deviance, we did not find any change over time for both groups, among all three conditions (repetitive letter sequences without speed specification, as fast as possible, and as precisely as possible), with one exception: Children of the waiting group wrote more coherent letters at t5 compared to t1 when writing repetitive letter sequences as fast as possible (z = −2.359, *p* = 0.018), whereas the children of the therapy group did not improve in this respect.

**Figure 3:**
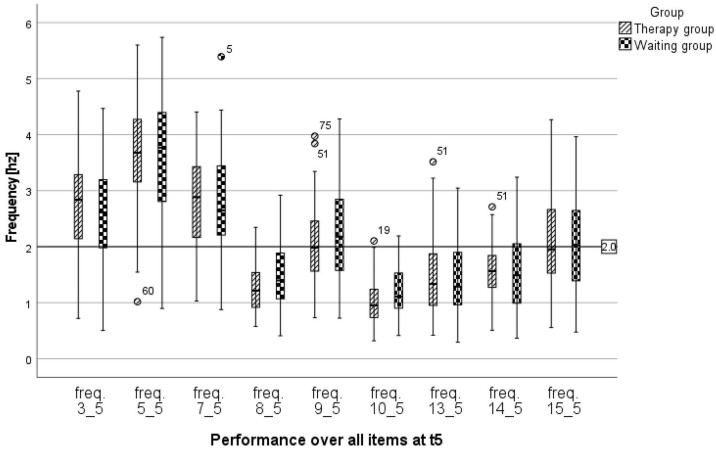
Performance regarding frequency over all items at t5 (freq.3 = Finger movements (fast); freq.5 = Wrist movements (fast); freq.7 = Combined finger and wrist movements (fast); freq.8 = Repetitive letter sequences (no speed specification); freq.9 = Repetitive letter sequences (as fast as possible); freq.10 = Repetitive letter sequences (as precisely as possible); freq.13 = Copying the word “neu”; freq.14 = Copying a sentence (no speed specification); freq.15 = Copying a sentence (fast). Notes: The bar at 2 Hz marks the threshold at which controlled movement changes to automated execution [17]. The numbers 60, 5, 51, 75, 19 denote the cases that are mild outliers outside the distribution.

Despite several improvements over time (as reported above), a narrower inspection of the performance at t5 made it clear that, depending on the task, the children did not achieve the target of sufficient frequency (i.e., a frequency of 2 Hz or greater). More than 75% of all children managed the basic movements in an automated manner, while in the remaining tasks 70–100% of the children were not able to do so (Figure 3).

### 3.3. Handwriting Self-Concept

Because some of the children did not answer all questions, the number of participants of each item differs slightly (range: 108–114 children).

Compared to the baseline assessment (t1), the children of the therapy group rated their handwriting as more beautiful (z = −2.70, *p* = 0.007), more legible (z = −2.24, *p* = 0.025), more skilful in terms of letter sequences (z = −2.26, *p* = 0.024), and were more satisfied with their handwriting (z = −2.41, *p* = 0.016) at t5. In contrast, the respective ratings did not change over time among the waiting group children. Regarding the remaining self-concept variables, the ratings of both groups did not change over time.

## 4. Discussion

We found a significant treatment effect with respect to fine motor control. PMT significantly improved fine motor skills in children with DCD and DD over a 5-month period compared to those in the waiting group regardless of diagnosis.

In contrast, we found no evidence that the treated children improved more than the waiting children (regardless of diagnosis DD or DCD) with respect to their graphomotor skills, such as fluency (frequency), automaticity, and consistency of forming letters over the 16-week period, but we did find several time effects for both groups.

In relation to the self-concept of handwriting, the treated children rated some aspects better at t5 compared to t1, while the ratings of the children of the waiting group remained stable over time.

The results regarding fine motor control are in line with the fact that PMT often starts to work on fine motor development as a precursor skill to writing by hand, e.g., the strengthening and mobility of the fingers is built up in this way [74]. The reasons for the increase in fine motor skills still need to be substantiated by the differentiated analysis of the protocol data, but they seem comprehensible. As the initial results of our therapy protocol analyses reveal [82], the focus was indeed on directly handwriting-related precursor motor skills such as pen posture and finger movement control (25% of the total therapy time). Additionally, children usually respond very well to the game-centred approach of PMT to promote fine motor skills. The approach superficially allows for attractive choices and seems less school-related and less performance-oriented than training graphomotor skills; therefore, it can be worked with a high level of intrinsic motivation, as recommended by the medical guidelines [64].

When it comes to the treatment of graphomotor difficulties, task-oriented therapy approaches are assumed to be more successful than process-oriented ones [58]. The initial results of our therapy protocol analysis [82] demonstrated that the pencil-and-paper-based promotion of visuomotor skills (11%) and direct handwriting training (15.5%) took up a quarter of all training units. Therefore, our zero finding regarding handwriting skills is unexpected, especially given the fact that even short-term task-based interventions improve handwriting fluency among struggling and typically developing young handwriters [99]. These findings may have several explanations.

First, the duration of some therapies did not reach the intended duration in the 16 weekly sessions, as there were failures due to illness, holidays, and school projects. With at least 10 sessions among the shortest interventions, this was just below the recommended threshold of guidelines. According to the meta study of Smits-Engelsmann et al. [58], most of the investigated interventions that were successful lasted longer than 10 weeks.

Second, during the intervention time, the pencil was used daily in class and explicit handwriting was taught and practiced several times a week. Therefore, the teaching effects probably suppressed the therapy effects.

Third, learning processes in handwriting are known to be non-linear; for example, children with ADHD initially show deterioration in their handwriting fluency when they obtain the perfect mix of medication and therapeutic treatment, at which time they are finally able to focus and learn, and therefore tend to write more slowly and in a less automated manner [100]. This could be the case here as well. When children learn to focus during the first therapy weeks, they will be ready to learn, even in terms of handwriting, but the increase will not be immediately visible. Fortunately, most of PMTs last longer, so some starting difficulties or even regressions can be absorbed. Due to ethical constraints, it was not possible to extend the duration of the study time for each cohort, even though the usual therapies last longer.

Furthermore, it is possible that these children were too young for a purely task-oriented approach to promoting graphomotor skills. Fine and gross motor movement opportunities, such as those available in the therapy room, are more in line with the fundamental need for play at this age. It is known that children who lacked play opportunities for a variety of reasons show a pronounced need to catch up [101] (p. 254), [102]. However, targeted work on handwriting corresponds less to the child’s intrinsic motivation but is usually a concern of parents and teachers. Therapists cannot resolve this conflict of goals; at most, they can steer it in a constructive direction by making agreements with the children, and they rely on the fact that playing creates an essential foundation for further development.

Regarding our data so far, we can conclude that all the children were making progress in terms of handwriting, but their performance at t5 was still far below the target range. As normally developed second graders can write common short words in a fast and almost automatised manner [103], the children in our sample showed a lower speed (below 2 Hz), indicating controlled fine motor steering even at t5 over all items that were more complex than basic movements. According to the cognitive load theory [104], this lack of automaticity is unfavourable, because children need to master basic handwriting and spelling skills in a fluent way to obtain more free resources in their working memory for the higher demands of writing.

The newly created DTW tasks unfortunately turned out to be too difficult for many children. Compared to the performance of a pilot study, many children of this sample of struggling handwriters were unable to write a sufficient number of valid letters in the given time or to reproduce non-recognizable or wrong letter forms. Additionally, due to the teaching material used in many classes, the children had surprisingly no experience with lowercase letters, not even with a common “a”. Therefore, our zero finding should be considered with caution. More research using a simpler task is necessary to come to a more comprehensive conclusion.

A comparison with the performance of normally developing second graders [103] can reveal areas in which children with DCD or DD still require therapy and whether this can lead to values in the target range in the longer term. Further research is necessary to analyse the outcomes of therapy possible with the therapy protocols, which are planned to be examined in detail next.

The finding that the children in therapy improved their self-concept more than the waiting group children is not surprising and is in line with our respective hypothesis, as PMT has an explicit focus on individual progress, which is discussed at several instances with the child [105,106]. Regarding the Reciprocal Effect Model [107], in which self-concept and performance influence each other, this improvement is important for the further course of therapy.

Finally, we emphasise that even regarding these limitations, the increase in a positive self-concept is a crucial first step to gain more joy in writing and motivation for a therapy that may simply take more time.

## 5. Conclusions

The present study successfully demonstrates that PMT improves fine motor skills, which are assumably prerequisites of handwriting, among young children, as well as improves some aspects of the handwriting self-concept, which is expected to support further learning and training. Thus, despite the short-term limitations of this study, our results provide scientific evidence for the current PMT services in Switzerland.

As handwriting acquisition implies complex learning processes, PMT can be considered a long-term endeavour. Our study children who attended PMT for half a year remain below the level of automatised handwriting movements and clearly need additional therapeutic support. However, given the limited results with respect to handwriting fluency and consistency, more research, taking longer time periods into account to observe respective improvements, is necessary.

### Limitations

Due to challenges associated with the pandemic, the data collection time was extended from two to three cohorts to meet the required sample size. Although there was no complete data loss, the precise rhythm of monthly examinations was difficult to fulfil, e.g., due to illnesses among the children, therapists, or teachers.

## Figures and Tables

**Table 1:** Description of the sample.

			Therapy Group		Control Group			
Variable	*n*	%	*n*	%	*n*	%	χ^2^	*p*
	121	100	61	50.4	60	49.6		
**Sex**							0.068	n.s.
-Female	31	25.6	15	24.6	16	26.7		
-Male	90	74.4	46	75.4	44	73.3		
**Handedness**							0.743	n.s.
-Righthanders	106	87.6	55	90.2	51	85		
-Lefthanders	15	12.4	6	9.8	9	15		
**Diagnosis**							0.752	n.s.
-DCD	42	34.7	22	36.1	20	33.3		
-DD	79	65.3	39	63.9	40	66.7		
**Class**							0.089	n.s.
-First graders	48	39.7	25	41	23	38.3		
-Second graders	73	60.3	36	59	37	61.7		

Notes: DCD = developmental coordination disorder; DD = developmental dysgraphia. n.s. = not significant.

**Table 2:** Items of handwriting measurement.

Item No.	Task	Item No.	Task
1	Scribbling (for trying out, not evaluated)	8	Repetitive letter sequences (writing at least 8 times the letter “a”, no speed specification)
2	Finger movements (no speed specification)	9	Repetitive letter sequences (writing at least 8 times the letter “a”, as fast as possible)
3	Finger movements (fast)	10	Repetitive letter sequences (writing at least 8 times the letter “a”, as precisely as possible)
4	Wrist movements (no speed specification)	11	Patterns (garlands)
5	Wrist movements (fast)	12	Patterns (double loops)
6	Combined finger and wrist movements when circling (no speed specification)	13	Copying the word “neu” (new) three times
7	Combined finger and wrist movements when circling (fast)	14	Copying a sentence “Die Kinder fliegen nach Amerika” (”The children fly to America”, no speed specification)
		15	Copying a sentence “Die Kinder fliegen nach Amerika” (“The children fly to America”, fast).

**Table 3:** Fine manual control (T-values) from t1 to t5 by group and diagnosis.

	Therapy Group		Control Group						
	DD	DCD	DD	DCD	Time	Group	Diagnosis	Time × Group	Time × Diagnosis
Measures	M	M	M	M	F	F	F	F	F
	(SD)	(SD)	(SD)	(SD)	df	df	df	df	df
					Eta^2^	Eta^2^	Eta^2^	Eta^2^	Eta^2^
					*p*	*p*	*p*	*p*	*p*
t1	39.4	31.65	37.41	33.79	2.699	7.19	7.563	28.74	3.082
	(1.43)	(1.89)	(1.41)	(1.96)	1/108	1/108	1/108	1/108	1/108
t5	45.81	40.38	34.98	34.64	0.024	0.062	0.065	0.21	0.028
	(1.49)	(1.96)	(1.46)	(2.04)	n.s.	0.008	0.007	0	0.082

Notes: The covariates in the model were calculated using the following values: Grade = 1.61 (n.s.). *n* = 113.

## Data Availability

The data presented in this study are openly available. Hurschler Lichtsteiner, Sibylle, Nideröst, Melanie, Wicki, Werner and Wyss, Stefanie (2023). Wirksamkeit Psychomotoriktherapie Grafomotorik (2019–2022) (1.0.0) [Dataset]. FORS Datenservice. https://doi.org/10.48573/gka9-vq56 (accessed on 1 May 2023).

## Appendix A

**Table A1:** Descriptive statistics of process-based handwriting: frequency and automaticity.

	t1			t2			t3			t4			t5		
	Total	Therapy Group	Waiting Group	Total	Therapy Group	Waiting Group	Total	Therapy Group	Waiting Group	Total	Therapy Group	Waiting Group	Total	Therapy Group	Waiting Group
Measures	M (SD)	M (SD)	M (SD)	M (SD)	M (SD)	M (SD)	M (SD)	M (SD)	M (SD)	M (SD)	M (SD)	M (SD)	M (SD)	M (SD)	M (SD)
FREQ 3	2.41 (0.99)	2.29 (0.94)	2.53 (1.03)	2.61 (0.96)	2.72 (0.97)	2.50 (0.95)	2.73 (1.06)	2.78 (1.02)	2.69 (1.12)	2.77 (0.97)	2.80 (0.94)	2.74 (1.02)	2.69 (0.83)	2.76 (0.82)	2.62 (0.85)
NIV 3	1.87 (1.05)	1.90 (0.99)	1.84 (1.28)	1.68 (0.94)	1.52 (0.78)	1.84 (1.06)	1.47 (0.77)	1.43 (0.82)	1.51 (0.69)	1.51 (0.94)	1.38 (0.65)	1.65 (1.17)	1.57 (1.52)	1.43 (0.96)	1.74 (1.99)
FREQ 5	3.44 (1.00)	3.60 (0.98)	3.26 (1.01)	3.71 (0.98)	3.86 (1.00)	3.55 (0.94)	3.69 (1.01)	3.73 (1.04)	3.65 (0.99)	3.68 (1.11)	3.79 (1.11)	3.58 (1.10)	3.67 (0.96)	3.70 (0.87)	3.64 (1.05)
NIV 5	1.26 (0.99)	1.21 (1.09)	1.31 (0.87)	1.12 (0.33)	1.13 (0.31)	1.11 (0.35)	1.11 (0.26)	1.10 (0.19)	1.12 (0.32)	1.51 (0.45)	1.17 (0.55)	1.13 (0.34)	1.10 (0.31)	1.09 (0.37)	1.11 (0.24)
FREQ 7	2.62 (0.96)	2.65 (0.98)	2.58 (0.95)	2.75 (0.96)	2.89 (0.96)	2.62 (0.95)	2.84 (0.90)	2.92 (0.81)	2.75 (0.98)	2.83 (0.97)	2.95 (0.92)	2.71 (1.01)	2.80 (0.88)	2.83 (0.81)	2.77 (0.94)
NIV 7	1.86 (1.58)	1.82 (1.55)	1.90 (1.63)	1.75 (1.73)	1.61 (1.55)	1.89 (1.89)	1.47 (0.83)	1.39 (0.85)	1.56 (0.81)	1.70 (1.78)	1.49 (1.20)	1.91 (2.20)	1.47 (0.84)	1.43 (0.64)	1.52 (1.02)
FREQ 8	1.12 (0.38)	1.20 (0.42)	1.06 (0.32)	1.33 (0.49)	1.32 (0.52)	1.34 (0.46)	1.32 (0.44)	1.24 (0.38)	1.42 (0.49)	1.42 (0.54)	1.35 (0.49)	1.50 (0.58)	1.38 (0.51)	1.27 (0.45)	1.50 (0.55)
NIV 8	5.47 (2.37)	5.06 (2.15)	5.86 (2.53)	4.57 (2.76)	4.85 (1.07)	4.26 (2.37)	4.13 (2.10)	4.41 (1.99)	3.82 (2.20)	4.05 (2.64)	4.38 (2.71)	3.71 (2.55)	4.17 (2.54)	4.50 (2.45)	3.79 (2.61)
FREQ 9	1.70 (0.54)	1.74 (0.55)	1.66 (0.54)	2.00 (0.68)	1.99 (0.71)	2.01 (0.65)	0.99 (0.39)	1.89 (0.74)	2.07 (0.76)	2.05 (0.76)	2.09 (0.77)	2.01 (0.76)	2.15 (0.76)	2.08 (0.69)	2.22 (0.83)
NIV 9	3.03 (1.67)	2.91 (1.54)	3.15 (1.81)	2.51 (1.49)	2.51 (1.48)	2.51 (1.52)	2.54 (1.52)	2.57 (1.30)	2.50 (1.77)	2.46 (1.69)	2.27 (1.23)	2.65 (2.06)	2.15 (1.12)	2.12 (1.06)	2.19 (1.20)
FREQ 10	0.89 (0.28)	0.90 (0.31)	0.87 (0.24)	1.09 (0.41)	1.10 (0.45)	1.07 (0.38)	0.99 (0.39)	0.95 (0.37)	1.05 (0.42)	1.07 (0.42)	0.98 (0.36)	1.16 (0.45)	1.10 (0.41)	1.01 (0.38)	1.21 (0.43)
NIV 10	8.03 (3.51)	8.09 (3.80)	7.98 (3.23)	6.22 (3.21)	6.24 (3.43)	6.19 (2.99)	7.11 (3.82)	7.29 (3.42)	6.89 (4.28)	6.53 (4.12)	7.30 (4.74)	5.71 (3.17)	6.22 (3.71)	6.97 (4.08)	5.40 (3.07)
FREQ 13	1.03 (0.45)	1.08 (0.44)	0.99 (0.46)	1.16 (0.56)	1.21 (0.57)	1.11 (0.56)	1.25 (0.56)	1.23 (0.54)	1.26 (0.60)	1.37 (0.63)	1.39 (0.59)	1.35 (0.67)	1.45 (0.63)	1.46 (0.63)	1.43 (0.65)
NIV 13	6.98 (5.99)	6.49 (6.20)	7.50 (5.76)	5.99 (4.98)	5.60 (4.43)	6.40 (5.49)	5.30 (4.03)	4.99 (3.06)	5.63 (4.85)	4.53 (3.68)	4.11 (2.79)	4.94 (4.37)	3.98 (3.44)	3.74 (2.53)	4.23 (4.17)
FREQ 14	1.15 (0.52)	1.15 (0.48)	1.14 (0.56)	1.36 (0.59)	1.37 (0.58)	1.34 (0.60)	1.46 (0.61)	1.46 (0.57)	1.45 (0.65)	1.57 (0.69)	1.60 (0.65)	1.56 (0.74)	1.57 (0.59)	1.57 (0.49)	1.57 (0.68)
NIV 14	5.41 (3.57)	5.20 (3.60)	5.64 (3.56)	4.50 (3.59)	4.36 (3.17)	4.64 (3.99)	3.97 (3.28)	3.67 (2.23)	4.27 (4.07)	3.42 (2.21)	3.17 (1.82)	3.68 (2.54)	3.32 (2.41)	3.04 (1.86)	3.61 (2.85)
FREQ 15	1.63 (0.68)	1.64 (0.71)	1.63 (0.65)	1.78 (0.72)	1.85 (0.76)	1.72 (0.68)	1.90 (0.80)	1.97 (0.81)	1.82 (0.78)	2.00 (0.84)	2.06 (0.77)	1.95 (0.90)	2.07 (0.86)	2.03 (0.82)	2.11 (0.90)
NIV 15	3.42 (2.58)	3.56 (2.93)	3.28 (2.18)	2.91 (2.01)	2.83 (2.06)	3.00 (1.96)	2.78 (2.22)	2.59 (1.96)	2.98 (2.46)	2.50 (1.76)	2.28 (1.50)	2.72 (1.97)	2.37 (1.57)	2.36 (1.61)	2.38 (1.55)

Notes: Descriptive statistics of writing characteristics. Legend: 3 = Finger movements (fast); 5 = Wrist movements (fast); 7 = Combined finger and wrist movements (fast); 8 = Repetitive letter sequences (no speed specification); 9 = Repetitive letter sequences (as fast as possible); 10 = Repetitive letter sequences (as precisely as possible); 13 = Copying the word “neu” freq.; 14 = Copying a sentence (no speed specification) freq.; 15 = Copying a sentence (fast). The sample varied because of invalid attempts: Item 3 range *n* = 59–100, Item 5 range *n* = 98–115, Item 7 range *n* = 105–116, Item 8 range *n* = 69–100, Item 9 range *n* = 77–100, Item 10 range *n* = 79–108; Item 13 range *n* = 113–120, Item 14 range *n* = 116–121, Item 15 range *n* = 91–114.

**Table A2:** Descriptive statistics of process-based handwriting: measures of dynamic time warping.

	t1			t2		t3			t4			t5			
	Total	Therapy Group	Waiting Group	Total	Therapy Group	Waiting Group	Total	Therapy Group	Waiting Group	Total	Therapy Group	Waiting Group	Total	Therapy Group	Waiting Group
Measures	M	M	M	M	M	M	M	M	M	M	M	M	M	M	M
	(SD)	(SD)	(SD)	(SD)	(SD)	(SD)	(SD)	(SD)	(SD)	(SD)	(SD)	(SD)	(SD)	(SD)	(SD)
DTW 8 Distance	0.08	0.08	0.09	0.08	0.08	0.09	0.08	0.08	0.08	0.08	0.08	0.08	0.08	0.08	0.08
	(0.04)	(0.03)	(0.04)	(0.33)	(0.03)	(0.04)	(0.03)	(0.03)	(0.03)	(0.08)	(0.02)	(0.04)	(0.07)	(0.3)	(0.03)
DTW 8 Consistency	35.57	37.95	33.26	29.81	31.92	27.48	29.91	32.09	27.49	30.81	32.92	28.59	31.33	34.11	28.19
	(22.44)	(21.99)	(22.95)	(24.04)	(20.43)	(20.24)	(17.63)	(19.11)	(15.73)	(21.02)	(21.97)	(20.03)	(19.47)	(20.14)	(18.39)
DTW 8	20.05	20.24	19.86	23.51	22.58	24.53	22.23	22.23	22.23	21.37	19.56	23.27	21.3	19.93	22.84
Deviance	(18.05)	(18.42)	(17.94)	(18.79)	(16.92)	(20.86)	(17.62)	(19.40)	(15.67)	(17.36)	(16.29)	(18.42)	(20.27)	(16.99)	(23.52)
DTW 9 Distance	0.10	0.09	0.10	0.09	0.09	0.09	0.10	0.10	0.10	0.10	0.09	0.10	0.10	0.09	0.10
	(0.05)	(0.04)	(0.05)	(0.04)	(0.03)	(0.04)	(0.04)	(0.04)	(0.40)	(0.04)	(0.03)	(0.04)	(0.03)	(0.03)	(0.30)
DTW 9 Consistency	26.17	27.25	26.17	23.89	24.63	23.89	20.13	23.84	20.13	20.19	22.85	20.19	19.19	22.58	19.19
	(18.21)	(21.5)	(18.21)	(18.24)	(18.7)	(18.24)	(16.01)	(19.00)	(16.01)	(15.83)	(18.14)	(15.83)	(14.67)	(18.42)	(14.67)
DTW 9	28.75	26.77	28.75	32.01	28.48	32.01	31.93	29.59	31.93	32.44	29.82	32.44	32.68	27.84	32.68
Deviance	(21.68)	(22.02)	(21.68)	(26.05)	(21.33)	(26.05)	(22.52)	(24.94)	(22.52)	(22.79)	(21.59)	(22.79)	(20.37)	(21.25)	(20.37)
DTW 10 Distance	0.10	0.08	0.10	0.09	0.08	0.09	0.08	0.08	0.08	0.09	0.08	0.09	0.08	0.07	0.08
	(0.05)	(0.04)	(0.05)	(0.04)	(0.03)	(0.04)	(0.05)	(0.04)	(0.05)	(0.04)	(0.04)	(0.04)	(0.03)	(0.03)	(0.03)
DTW 10 Consistency	25.76	35.25	25.76	29.16	31.54	29.16	29.61	32.66	29.61	25.96	34.9	25.96	29.13	36.53	29.13
	(19.27)	(24.94)	(19.27)	(17.79)	(23.39)	(17.79)	(19.88)	(21.50)	(19.88)	(16.00)	(22.07)	(16.00)	(17.75)	(20.76)	(17.75)
DTW 10	27.46	22.47	27.46	25.71	17.72	25.71	20.08	19.89	20.08	24.23	19.06	24.23	23.79	16.32	23.79
Deviance	(23.44)	(19.18)	(23.44)	(19.92)	(16.05)	(19.92)	(22.23)	(20.57)	(22.23)	(19.8)	(19.38)	(19.8)	(18.97)	(15.98)	(18.97)

Notes: Descriptive statistics of DTW writing characteristics. Legend: 8 = Repetitive letter sequences (no speed specification); 9 = Repetitive letter sequences (as fast as possible); 10 = Repetitive letter sequences (as precisely as possible). The sample varied because of invalid attempts: Item 8 range *n* = 69–100, Item 9 range *n* = 77–100, Item 10 range *n* = 79–108.
